# Cost analysis of HIV treatment and drug-related adverse events when fixed-dose combinations of antiretrovirals (FDCs) were stopped, versus continuation with FDCs

**DOI:** 10.1186/2191-1991-2-16

**Published:** 2012-09-03

**Authors:** Francesc Homar, Virginia Lozano, Juan Martínez-Gómez, Itziar Oyagüez, Antonio Pareja, Antoni Payeras, Joaquín Serrano, Carmen Carratalá, Miguel Ángel Casado

**Affiliations:** 1 Department of Internal Medicine, Son LLàtzer Hospital, Ctra. Manacor km 4, Palma de Mallorca, 07198, Spain; 2 Pharmacoeconomics and Outcomes Research Iberia (PORIB), Calle de la Golondrina, 40A, Madrid, 28023, Spain; 3 Department of Epidemiology, Son Llàtzer Hospital, Ctra. Manacor km 4, Palma de Mallorca, 07198, Spain

**Keywords:** HIV, Fixed-dose combination, Antiretrovirals, Cost-analysis, Spain

## Abstract

**Background:**

The lower sales price of generic lamivudine has caused healthcare administrators to consider abolishing fixed-dose antiretroviral combinations (FDCs) that contain lamivudine and emtricitabine. The alternative is to administer the individual components of the FDCs separately, thus incorporating the new generic lamivudine medication.

**Methods:**

The Balearic Islands Health Service ordered the discontinuation of the treatment with FDCs in July 2010, but FDCs were reintroduced in August 2010. At that point, an independent, retrospective cost analysis was performed by Son Llàtzer Hospital. A total of 75 patients who were treated from July to August 2010 underwent replacement of their FDC treatment with the individual components. Additionally, 150 patients who continued using FDCs were randomly selected. For both patient groups, the antiretroviral therapy that was administered and the costs associated with management of adverse events were recorded. The study period used for the cost calculations was the average number of days that patients used separate components of FDCs (120 days). An alternative analysis was performed to consider the costs of the extra follow-up visit (consultation and clinical tests) that was required for patients who changed their antiretroviral therapy.

**Results:**

Considering antiretroviral therapies and adverse events, the administration of the separate components increased the total daily cost by 0.72 € per patient compared to treatment with FDCs. When the cost of an extra follow-up visit was considered, the daily cost increased by 3.61 € per patient.

**Conclusions:**

Our study suggests that the discontinuation of FDC treatment and the replacement with the administration of separate antiretroviral agents could lead to an increase in healthcare costs due to the higher rate of adverse events that was observed with the discontinuation of FDCs.

## Background

Human immunodeficiency virus (HIV) is a retrovirus of the *Lentivirus* genus. The infection produced by HIV leads to the development of the acquired immunodeficiency syndrome (AIDS). HIV preferentially attacks CD4+ cells, a type of T lymphocyte, which prevents the immune system from reacting to opportunistic infections that are produced by other viruses, bacteria, or fungi. According to the latest World Health Organisation (WHO) data in December 2011, the number of people infected by HIV is estimated to be 34 million [1]. In 2010, 1.8 million people died as a consequence of illnesses related to AIDS, which makes this pandemic disease the fourth leading cause of mortality worldwide [1].

Because of the complexity associated with the selection of a specific treatment, the severity of the related side effects and the necessity of preventing the appearance of resistant viral strains, the current treatment of HIV-1 is based on a combination of antiretroviral agents. This therapy, known as “highly active antiretroviral therapy” (HAART), has evolved over the years from a regimen of more than 20 pills per day to a single daily pill that combines fixed doses of several antiretroviral agents [2].

Antiretroviral therapy (ART) should be administered indefinitely to patients infected with HIV-1. The therapy, which has possible short- and long-term side effects, requires constant therapeutic compliance to complex drug regimens. Fixed-dose antiretroviral combinations (FDCs) have led to the simplification of antiretroviral therapy, which has improved patient quality of life and treatment compliance [3].

Due to the current economic crisis and budget restrictions, the arrival of generic lamivudine with a more competitive sales price has led some healthcare administrators to consider breaking up FDCs that contain lamivudine (3TC) and emtricitabine (FTC), and administrating the separate components that includes the new generic lamivudine. FDCs containing emtricitabine, such as Atripla^TM^ (efavirenz, emtricitabine, and tenofovir) and Truvada^TM^ (emtricitabine and tenofovir), have also been subject to interruptions in favour of the administration of the separate components. In these cases, the emtricitabine component was substituted with generic lamivudine.

To estimate the effect of this decision at Son Llàtzer Hospital, a retrospective study was performed to analyse information about the administration of ART and the healthcare resources that are used to manage adverse events (AEs). In the present study, an analysis of the ART and AEs associated costs was performed for patients treated with FDCs and for patients treated with combinations of separately administered antiretrovirals.

## Methods

**Figure 1:**
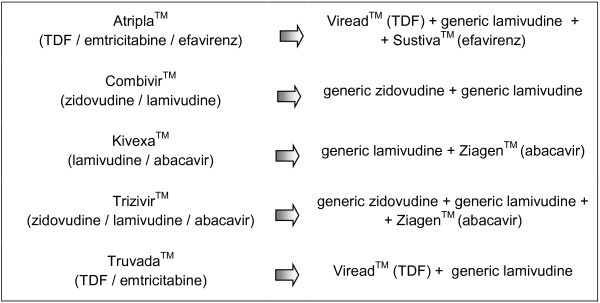
Schematic FDCs separation to individual antiretroviral agents.

An independent, retrospective study was conducted at Son Llàtzer Hospital between June 2010 and July 2011. Information regarding ART and the management of AEs was collected from patients infected with HIV-1. A total of 75 patients experienced the substitution of their FDC treatment with individual antiretroviral agents (exposed group). The schematic in Figure 1 represents how the FDCs were breaking down. The medications Combivir^TM^ (lamivudine and zidovudine), Kivexa^TM^ (abacavir and lamivudine), and Trizivir^TM^ (abacavir, lamivudine, and zidovudine) were replaced with the separate administration of their individual antiretroviral agents; generic lamivudine for Kivexa^TM^ and generic zidovudine and lamivudine for Combivir^TM^ and Trizivir^TM^. In contrast, and because generics are still unavailable for the components of Atripla^TM^ (efavirenz, emtricitabine, and tenofovir) and Truvada^TM^ (emtricitabine and tenofovir) emtricitabine was replaced with generic lamivudine, which is perceived to be equivalent to emtricitabine.

Using a hospital database of 434 patients with controlled HIV, 150 patients observed between March and November 2010, and who continued receiving FDCs, were randomly selected (non-exposed group); these patients continued receiving FDCs because their expected appointment dates preceded the FDCs disruption. The following baseline characteristics were recorded for both group of patients: gender, age, weight, date of HIV diagnosis, mechanism of transmission, history of AIDS, HCV infection status, psychiatric history, current use of methadone or psychopharmaceuticals, date of efavirenz initiation, date of FDC initiation and type, non-nucleoside analogue reverse transcriptase inhibitor use, protease inhibitor use, and integrase inhibitor use (Table 1).

**Table 1:** Baseline patient characteristics

	**Exposed patients (n = 75)**	**Non-exposed patients (n = 150)**	
Gender (female)	18 (24%)	38 (25%)	*
Mean age (years)	44	46	*
Weight (kg)	70	72	*
**Mechanism of transmission**			*
Homosexual	18 (25%)	37 (25%)	*
IVDU	29 (39%)	52 (35%)	*
Heterosexual	19 (25%)	48 (32%)	*
Unknown	7 (9%)	12 (8%)	*
**Concomitant pathologies**			*
AIDS	18 (24%)	46 (30%)	*
HCV	31 (40%)	56 (39%)	*
Psychiatric history	17 (23%)	27 (18%)	*
Methadone consumption	5 (6%)	22 (14%)	*
Use of psychopharmaceuticals	14 (19%)	19 (13%)	*
**FDCs**			*
Atripla^TM^	36 (48%)	72 (48%)	*
Truvada^TM^	24 (32%)	48 (32%)	*
Kivexa^TM^	7 (9%)	16 (11%)	*
Combivir^TM^	6 (8%)	14 (9%)	*
Trizivir^TM^	2 (3%)	0 (0%)	*
**NNRTI**	15 (20%)	28 (19%)	*
**PI**	22 (29%)	54 (36%)	*
**Integrase**	0 (0%)	3 (2%)	*
Exposure time to FDCs (months)	27 (range: 1–121)	24 (range: 3–98)	*
Exposure time to efavirenz (months)	50 (range: 129–1)	61 (range: 126–3)	*
Viral load > 50 copies/mL at visit −1	12 of 74 (16.2%)	8 of 149 (5.4%)	(OR: 3.4; 95%CI: 1.3-8.8; p = 0.02)
Viral load > 50 copies/mL at baseline visit	10 of 74 (13.5%)	10 of 146 (6.8%)	*
CD4 at first visit (cells/μL)	573 (range: 41–1527)	542 (range: 92–1481)	*

IVDU: intravenous drug user; AIDS: acquired immunodeficiency syndrome; HCV: hepatitis C virus; FDCs: fixed-dose antiretroviral combinations; NNRTI: non-nucleoside reverse transcriptase inhibitor; PI: protease inhibitor; CD4: CD4+ lymphocytes; OR: odds ratio; 95%CI: confidence intervals at the 95% level.

*No statistically significant differences.

For the exposed group, the baseline visit was defined as the date on which the FDC change was performed (between July and the beginning of August 2010). Data were also collected from the visit prior to the baseline visit (visit −1) and from two serial visits after the baseline visit (visits +1 and +2). For the non-exposed patients, the baseline visit was the closest visit to July 2010. At each visit, the CD4+ T lymphocyte counts, HIV-1 viral load, serum glutamic oxalacetic transaminase (SGOT), serum glutamic pyruvate transaminase (SGPT), cholesterol, LDL-cholesterol, HDL-cholesterol, triglycerides, and the presence of AEs were recorded for both group of patients. During the process of identifying AEs, the potential relationship with ART was indicated (unrelated or likely related). This decision was based on the investigator’s criteria and on the attending physician’s annotations in the patient’s chart (the temporal relationship and the disappearance or improvement of the AE after withdrawing from the antiretroviral agent). The severity of AEs was recorded according to the World Health Organization criteria. The possible reintroduction of the FDCs on visits +1 and +2, changes to other treatments, and failures to follow-up were also recorded.

The average time that the exposed patients were treated with individual antiretroviral agents (120 days) was used as a temporal horizon for the cost analysis. Given that this analysis was performed from a hospital perspective, only healthcare costs that were directly associated with the pharmaceutical cost and the cost of managing the AEs were included. For the cost analysis, only AEs that were related to ART were considered. The consumption of antiretroviral agents was calculated using the posology from the corresponding Summary of Product Characteristics, and considering the ex-factory price that results from converting the catalogue price of the Spanish General Council of Official Colleges of Pharmacists [4] with the established commercial margins [5] (Table 2). The cost of AEs associated with ART was calculated based on the resource utilisation during AE management. The unit costs of the healthcare resources were obtained from a Spanish database of healthcare costs [6] (Table 3).

**Table 2:** Unit costs of antiretroviral agents

**Antiretroviral agent**	**Cost (€)**
**FDCs**	
Atripla^TM^ (TDF / emtricitabine / efavirenz: 245 / 200 / 600 mg), 30 tablets	701.08
Combivir^TM^ (zidovudine / lamivudine: 300 / 150 mg), 60 tablets	290.41
Kaletra^TM^ (lopinavir / ritonavir: 200 / 50 mg), 120 tablets	400.02
Kivexa^TM^ (lamivudine / abacavir: 300 / 600 mg), 30 tablets	355.54
Trizivir^TM^ (zidovudine / lamivudine / abacavir: 300 / 150 / 300 mg), 60 tablets	490.32
Truvada^TM^ (TDF / emtricitabine: 200 / 245 mg), 30 tablets	432.73
**Individual antiretroviral components of FDCs**	
Generic lamivudine (300 mg), 30 tablets	62.76
Sustiva^TM^ (efavirenz: 600 mg), 30 tablets	265.03
Viread^TM^ (TDF: 250 mg), 30 tablets	288.70
Ziagen^TM^ (abacavir: 300 mg), 60 tablets	225.69
Generic zidovudine (300 mg), 300 tablets	403.15
**Other antiretroviral agents employed in addition to FDCs**	
Intelence^TM^ (etravirine: 100 mg), 120 tablets	450.00
Isentress^TM^ (raltegravir: 400 mg), 60 tablets	690.00
Norvir^TM^ (ritonavir: 100 mg), 30 tablets	22.46
Prezista^TM^ (darunavir: 400 mg), 60 tablets	427.21
Reyataz^TM^ (atazanavir: 200 mg), 60 tablets	436.59
Reyataz^TM^ (atazanavir: 300 mg), 30 tablets	436.59
Telzir^TM^ (fosamprenavir: 700 mg), 60 tablets	316.89
Viramune^TM^ (nevirapine: 200 mg), 60 tablets	199.69

Ex-factory price (€, 2011).

**Table 3:** Unit costs of healthcare resources (€, 2011)

**Healthcare resource**	**Unit cost (€)**
**Consultations**	
Mental health day centre	49.36
Infectious disease (subsequent consultation)	114.82
Nursing	15.69
Emergency	160.14
**Admissions**	
Internal medicine	526.28
Neurology	410.23
Intensive care unit	1,797.37
**Procedures**	
Liver biopsy	389.73
Colonoscopy	200.80
Abdominal ultrasound	82.61
Breast ultrasound	47.04
Electroencephalogram	77.94
Lumbar puncture	269.90
Cerebral MRI	232.38
Chest X-ray	20.94
Cranial computer tomography	109.12
**Laboratory studies**	
Urinalysis	12.07
Biochemistry	26.38
Viral load	119.52
Lymphocyte subpopulation study	60.97
Coagulation profile	10.95
Liver profile	4.69
Serology	26.39
Erythrocyte sedimentation rate	1.12

### Alternative scenario

In the exposed group, as a consequence of the change in ART experienced with the replacement of their FDCs, the Son Llàtzer Hospital scheduled one follow-up visit for these patients. The costs related to this follow-up visit were included in an alternative scenario and entail the cost associated with the consumption of the following healthcare resources: a medical appointment, biochemistry, haemogram, coagulation profile, urinalysis, analysis of lymphocyte populations, and determination of the viral load. The costs considered in this analysis are expressed in Euros (€) from 2011, and are detailed in Table 3. The study protocol was approved by the Research Commission of the Son Llàtzer Hospital. Written informed consent was obtained from the patients prior to data collection.

## Results

The mean age of the patients in the exposed group (n = 75) was 44 years, and in the non-exposed group (n = 150), 46 years. The percentage of women was similar in both groups of patients, with 24% in the exposed group and 25% in the non-exposed group. There were no statistically significant differences in the baseline patient characteristics of the exposed and non-exposed group, including patient weight, mechanism of HIV transmission, concomitant pathology, and type of FDC used at the beginning of the study (Table 1). At visit −1, 12 of 74 (16.2%) exposed patients and 8 of 149 (5.4%) non-exposed patients had HIV-1 viral load greater than 50 copies/mL (OR 3.4; 95%CI: 1.3-8.8; p = 0.02). At the baseline visit, the HIV-1 viral load was greater than 50 copies/mL in 10 of 74 (13.5%) exposed patients and in 10 of 146 (6.8%) non-exposed patients. At visit +1, 5 of 67 exposed patients (7.4%) presented viral load values greater than 50 copies/mL, whereas 13 of 146 non-exposed patients (8.9%) presented viral load values greater than 50 copies/mL; at visit +2, viral load values greater than 50 copies/mL were observed in 9 of 72 exposed patients (12.5%) compared with 9 of 134 non-exposed patients (6.7%). Except the HIV-1 viral load at visit −1, none of the other differences were statistically significant, and neither was the differences found in the CD4+ cell counts.

In total, 21 AEs (28.0%) were observed in the exposed group, and 7 AEs (4.7%) were observed in the non-exposed group. Of the AEs that were likely related to ART, there were 14 (18.7%) in the exposed group at visit +1, immediately after discontinuation with the FDC, compared with 2 (1.3%) in the non-exposed group (OR 16.8; 95%CI: 3.7-76.9; p  < 0.001). It should be noted that the 14 AEs observed in the exposed group were found in patients who were initially under stable treatment with an emtricitabine containing FDCs, Atripla^TM^ or Truvada^TM^, (n = 60).No AEs were found in the exposed patients that were previously treated with a lamivudine containing FDCs, Kivexa^TM^, Combivir^TM^ or Trizivir^TM^, (n = 15).

The differences on AEs likely related with ART at visit +1 between both groups of patients were extensively studied. When the AEs were analysed excluding those patients with HIV-1 viral load greater than 50 copies/mL at visit −1, statistically significant differences were also found. In the exposed patients with viral load lower than 50 copies/mL at visit −1 (n = 62), 11 (17.7%) AEs likely related to ART were found, while only 2 (1.4%) AEs were observed in the non-exposed group of patients (n = 141) (OR 14.9; 95%CI: 3.2-70.0; p  < 0.001). In addition, another analysis excluding patients with HIV-1 viral load > 50 copies/mL at baseline visit was carried out. There were found 13 (20.3%) AEs likely related with ART at visit +1 in 64 exposed patients compared with 2 (1.5%) AEs found in 136 non-exposed patients (OR 17.0; 95%CI: 3.7-78.3; p  < 0.001).

Another analysis was performed excluding those patients who recently started treatment with FDCs (less than 6 month of treatment with FDCs at the beginning of the study). In the exposed group of patients (n = 63), 11 (17%) AEs likely related with ART were observed at visit +1 compared with 1 (0.7%) AEs in the non-exposed group (n = 132) (OR 27.1; 95%CI: 3.4-215.8; p  < 0.001). Statistically significant differences were also observed when the patients who recently started treatment with efavirenz (less than 6 months) were excluded. In the exposed group (n = 50), 12 (24.0%) AEs were found compared with 2 (2.4%) AEs in the non-exposed group (n = 84) (OR 13.5; 95%CI: 2.9-63.5; p  < 0.001). The results of these analyses are summarized in Table 4.

**Table 4:** Adverse Events that are likely related to antiretroviral therapy at visit +1

	**Non-exposed (NE) group**	**Exposed (E) group**	
Total number of AEs found NE group: n = 150; E group: n = 75	2 (1.3%)	14 (18.7%)	OR 16.8; 95% CI: 3.7-76.9; p < 0.001
Number of AEs excluding patients with viral load > 50 copies/mL at visit −1 NE group: n = 141; E group: n = 62	2 (1.4%)	11 (17.7%)	OR 14.9; 95% CI: 3.2-70.0; p < 0.001
Number of AEs excluding patients with viral load > 50 copies/mL at baseline visit NE group: n = 136; E group: n = 64	2 (1.5%)	13 (20.3%)	OR 17.0; 95% CI: 3.7-78.3; p < 0.001
Number of AEs excluding patients who recently started treatment with FDCs NE group: n = 132; E group: n = 63	1 (0.8%)	11 (17.5%)	OR 27.1; 95% CI: 3.4-215.8; p < 0.001
Number of AEs excluding patients who recently started treatment with efavirenz NE group: n = 84; E group: n = 50	2 (2.4%)	12 (24.0%)	OR 13.5; 95% CI: 2.9-63.5; p < 0.001

*AEs: adverse events; NE: non-exposed; E: exposed: FDCs: fixed-dose antiretroviral combinations; OR: odds ratio; 95%CI: confidence intervals at the 95% level.

In the group of non-exposed patients, the two observed AEs that were likely related to ART, were neuropsychiatric effects and were also likely related to efavirenz toxicity. One of these patients presented with dizziness after making an error in taking Atripla^TM^, while the other patient presented with insomnia, anxiety, and abnormal dreams (Table 5).

**Table 5:** Description of adverse events that are likely related to antiretroviral therapy

**Adverse events**	**Severity (grade)**	**Resources consumed for the management of the adverse event**
*Non-exposed group (n = 150)*		
1. Dizziness after an error in taking Atripla^TM^	1	-
2. Neuropsychiatric: insomnia, anxiety, and abnormal dreams*	1	-
*Exposed group (n = 75)*		
1. Neuropsychiatric: dizziness	2	-
2. Neuropsychiatric: insomnia, abnormal dreams*	2	-
3. Neuropsychiatric: depression, insomnia*	1	-
4. Neuropsychiatric: insomnia	2	Infectious disease consult
5. Diarrhoea	2	-
6. Vomiting	2	-
7. Hepatic toxicity	4	Specialist and nursing consults, hospital admissions, chest x-rays, abdominal ultrasound, and hepatic biopsy
8. Neuropsychiatric: dizziness, insomnia, and abnormal dreams*	2	-
9. Neuropsychiatric: insomnia, anxiety, loss of concentration, abnormal dreams, and HIV encephalopathy after stopping treatment*	4	Infectious disease consults, hospital admissions, cranial CAT scan, cerebral MRI, and lumbar puncture
10. Neuropsychiatric: dizziness	1	-
11. Psychiatric: depression, insomnia, anxiety, abnormal dreams, and hallucinations*	3	Emergency department admission, mental health consult
12. Hepatic toxicity	3	Infectious disease consult, nursing, biochemistry, serology, and abdominal ultrasound
13. Neuropsychiatric: dizziness, insomnia, headache, and abnormal dreams*	2	Infectious disease consult
14. Diarrhoea	2	-

*Patients with more than one type of neuropsychiatric adverse event. The grade of the more severe AE is specified.

Of the 14 exposed patients who presented AEs that were likely related to ART, 9 patients who had undergone prior treatment with Atripla^TM^ presented with neuropsychiatric symptoms that were compatible with efavirenz toxicity, despite undergoing stable treatment with this agent; the mean time of exposure to efavirenz was 77 months in the patients with neuropsychiatric AEs and 44 months in the patients without neuropsychiatric AEs (p = 0.01). There were two other cases of neuropsychiatric AEs in the exposed group, but they were considered to be unrelated to ART due to the absence of a suggestive temporal relationship. Additionally, there were two cases of hepatic toxicity, two cases of diarrhoea, and one case of vomiting (Table 5).

In the exposed group, with respect to the severity of the AEs that were likely related to ART, there were two grade-4 AEs, including one case of toxic hepatitis and one neuropsychiatric event that included virologic failure and HIV encephalopathy. Additionally, there were two grade-3 AEs, including one hepatotoxicity AE and one neuropsychiatric AE. In these severe grade-3 and grade-4 AEs, the neuropsychiatric events were found in patients previously treated with Atripla^TM^, while the cases of hepatotoxicity were found in one patient who had been previously treated with Truvada^TM^ and fosamprenavir, and in other patient who had undergone previous treatment with Atripla^TM^.

**Figure 2:**
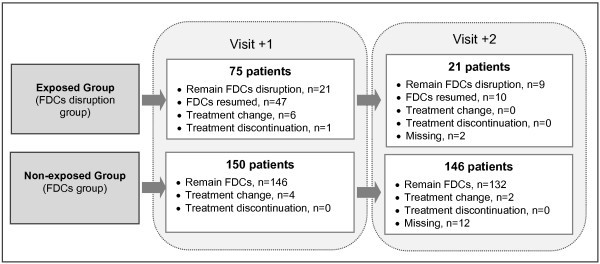
Flow of exposed and non-exposed patients during visits +1 and +2.

In Figure 2, the flow of exposed and non-exposed patients during visits +1 and +2 is described.

In the exposed group, FDCs were reintroduced on visit +1 in 47 (63%) patients. In 8 (12.6%) of these patients, the FDCs were introduced after the appearance of AEs that were likely related to ART. There were 6 (8%) patients who received a new FDC in the exposed group, and there were 4 (2.6%) patients who received a new FDC in the non-exposed group. On visit +2, 21 exposed patients continued with individual antiretroviral agents, and FDCs were reintroduced in 10 of these patients. The mean time that patients in the exposed group were treated with individual components of FDCs was 120 days. In the non-exposed group, the average cost per patient was 3,017.50 € during the study period, while the average cost per patient was 3,103.84 € in the exposed group (Table 6). The greater average antiretroviral treatment cost in the exposed group was due to the higher frequency of AEs in this group of patients. Of the 14 AEs that were likely related to ART in the exposed group, 6 AEs were related to the consumption of healthcare resources. The management of adverse events in the exposed group represented an additional cost of 230.26 € per patient compared with the non-exposed group. In the non-exposed group, the management of the two observed AEs involved no additional costs. Nevertheless, in the non-exposed group, the pharmaceutical cost per patient (3,017.50 €) was higher than that in the exposed group (2,873.58 €). Thus, considering the costs of ART and those associated with AEs management, administration of individual antiretroviral agents rather than FDCs increases the cost of daily treatment by 0.72 € per patient.

**Table 6:** Analysis of cost per patient (€, 2011)

	**Non-exposed group**	**Exposed group**	**Difference**
**Base case**			
Cost of ART during the study period*	3,017.50	2,873.58	143.92
Cost of AEs during the study period	0.00	230.26	−230.26
Average total cost during the study period	3,017.50	3,103.84	−86.34
Total cost per day	25.22	25.94	−0.72
**Alternative scenario**			
Cost of ART during the study period	3,017.50	2,873.58	143.92
Cost of AEs during the study period	0.00	230.26	−230.26
Cost of extra follow-up visit during the study period	0.00	345.83	−345.83
Total cost during the study period	3,017.50	3,449.67	−432.17
Total cost per day	25.22	28.83	−3.61

*Study period = 120 days; ART: antiretroviral therapy; AEs: adverse events.

The pharmaceutical cost has the greatest effect on the total cost of the treatment. The pharmacological cost represents 95% and 100% of the total cost in the exposed and non-exposed patient groups, respectively.

### Alternative scenario

The additional follow-up visit for patients in the exposed group incurred an added cost of 345.83 € per patient during the study period. Thus, considering the cost of an extra follow-up visit, the total daily cost of the treatment increased by 3.61 € per patient when antiretroviral agents were administered separately rather than using FDCs. In Table 6, the results are detailed for the base case and for the alternative scenario.

## Discussion

The lower cost of the treatment with separated individual antiretroviral agents rather than FDCs is associated with lower pharmaceutical costs but is related to higher total healthcare costs.

In 2010, 25 years had passed since the initiation of research on active compounds against HIV replication. During this time period, considerable advances were made. Possibly, there is no other therapeutic field that has obtained comparable results in such a short period of time. The advances include the development of FDCs that have improved the quality of life of patients with HIV by facilitating patient compliance and adherence to complex and life-long treatments [7]. Contrary to other chronic diseases, irregular compliance to treatments for HIV-1 infection can result in a definitive loss of efficacy of the therapeutic regimen and can lead to the development of resistance to the antiretroviral agents that are used for the treatment of the disease [8].

Emtricitabine and lamivudine are nucleoside analogue reverse transcriptase inhibitors, also known as “XTC” drugs. These two antiretroviral agents are similar in terms of structure, antiretroviral activity, and selection for the M1841/V mutation, which alone can compromise the response to both drugs. As a result, there is a perception that emtricitabine and lamivudine are equivalent and interchangeable. However, the replacement of emtricitabine, which is present in some FDCs, with generic lamivudine may be therapeutically non-equivalent. In this sense, recent studies suggest that combinations of antiretrovirals with lamivudine may be associated with higher rates of the M1841/V mutation than combinations with emtricitabine [9,10]. Maserati *et al.* suggested that this different rate of resistance to mutations may be the result of the different pharmacokinetic properties of lamivudine and emtricitabine [11].

In some recent studies, better results have been observed in the achievement of virologic response in patients treated with FDCs at risk of poor compliance, and other studies have reported a lower risk of hospital admission and less consumption of healthcare resources in patients treated with FDCs that consist of a single tablet per day [12,13]. While these studies do not demonstrate a cause-effect relationship, they do suggest that FDCs could increase the efficacy of ART and generate long-term savings in healthcare costs. In our study, we found a significant increase in AEs after break-up of the FDCs, which was associated with a short-term increase in healthcare costs.

As our study was based on clinical practice, all of the exposed patients were aware of the change and were most likely not in agreement with it; thus, we cannot discard the possibility that the AEs were caused by a placebo effect, especially in the milder cases. Nevertheless, this does not detract from the repercussions on daily clinical practice. Interestingly, the exposed patients who presented AEs were those who were under stable treatment with an emtricitabine containing FDCs prior to the treatment change. None of the exposed patients with a previous lamivudine containing FDCs presented any AEs after the FDCs break-up. Thus, the higher rate of AEs in the exposed group it could be attributed to the change of emtricitabine for lamivudine. However, these results should be interpreted with caution as the number of exposed patients from lamivudine containing FDCs was low (n = 15) and because in general, lamivudine is associated with few AEs.

In our study, there were no statistically significant differences between both groups of patients in variables such as age, gender, weight, mechanism of transmission, concomitant pathologies and type of ART, what otherwise could have explained the higher rate of AEs in the exposed group. The only statistically significant difference between exposed and non-exposed patients was the HIV-1 viral load at visit −1 (prior to baseline visit), but when a reanalysis was carried out excluding those patients, the differences found in AEs appearance continued to be significant. At baseline visit, there were also more patients with HIV-1 viral load > 50 copies/mL in the exposed group compared with the non-exposed group, without being this difference significant. When those patients were excluded from the analysis, the difference in AEs between both groups remained statistically significant. Therefore, it seems unlikely that the poorer control of the viral load in the exposed patients could be the reason for the higher rate of AEs observed in this group. Patients who recently started with ART have also more probabilities of present AEs. Thereby, reanalyses were carried out excluding patients with less than 6 months under treatment with FDCs or efavirenz, maintaining the differences in the AEs rate between both groups of patients.

Among the AEs, neuropsychiatric events occurred most frequently, which was similar to what was observed at the initiation of efavirenz treatment, regardless of whether the patients had been undergoing stable treatment. Studies have demonstrated a relationship between the presence of specific polymorphisms in cytochromes CYP2B6, CYP2A6, and CYP3A4 and a higher risk of neuropsychiatric toxicity that is associated with the discontinuation of efavirenz [14]; however, there is no evidence of a different interaction of lamivudine and emtricitabine with efavirenz. In contrast, in a study published by Pollock *et al.*, mild neuropsychiatric toxicity was found in some patients who switched from lamivudine to emtricitabine [15].

Although there are no data that demonstrate greater toxicity of lamivudine compared to emtricitabine [16], these AEs were clearly associated with FDCs disruption. The severe AEs, which had significant cost repercussions, were neuropsychiatric in three cases and hepatotoxic in two cases.

No significant differences were found in the rate of virologic failure between patients exposed and patients not exposed to FDCs disruption. However, the number of patients included in this study was small, the time of exposure to the change was short (an average of 120 days), and the majority of patients returned to using FDCs when their reintroduction was allowed.

Finally, it should be noted that the regional approval to separately administer FDCs components implies the access to different levels of healthcare quality according to the geographic area of residence, despite the availability of a common National Healthcare System in Spain. One methodological limitation of this cost analysis is that it was based on a single study centre and was not randomised. Nevertheless, the data recorded during this study reflect usual clinical practices. Efficacy parameters for different strategies have not been considered, as the objective of this study was not to perform a cost-effectiveness analysis; rather, the objective of the study was to analyse the possible savings that are associated with FDCs discontinuation in Son Llàtzer Hospital.

## Conclusions

The results of this study suggest that breaking up FDCs to separately administer the individual antiretroviral agents leads to an increase in costs for the Son Llàtzer Hospital. These additional costs were related to the management of AEs and the additional follow-up visits that require a change in ART in our hospital.

## Competing interests

This work was supported by an unrestricted research grant sponsored by Gilead. The authors have not transmitted any conflicts of interest, because the concept, design and development of the model have been carried out independently. VL, IO and MAC are PORIB employees a consultant company specialized in economic evaluation of health technologies.

## Authors’ contributions

FH and MAC conceived of the study and performed a general coordination of the project. VL and IO have made substantial contributions to design of the study, analysis, interpretation of the results and were involved in drafting the manuscript. FH, JMG, APareja, APayeras, JS and CC have played a key role in acquisition of data and validating the assumptions taken in the study design. All the authors have participated sufficiently in the work to take public responsibility for appropriate portions of the content. All of them have reviewed the final version of the manuscript and have given a final permission of the version to be published.
